# Roles of neuroimaging markers and biomarkers in cerebral small vessel disease and their associations with cognitive function

**DOI:** 10.3389/fneur.2025.1483842

**Published:** 2025-05-01

**Authors:** Xiaoqian Song, Chenyang Jin, Moxin Luan, Xueping Zheng

**Affiliations:** ^1^The Affiliated Hospital of Qingdao University, Qingdao, China; ^2^Qingdao Medical College, Qingdao University, Qingdao, China

**Keywords:** cerebral small vessel disease, neuroimaging markers, cerebrospinal fluid biomarkers, blood biomarkers, cognitive function

## Abstract

Cerebral small vessel disease (CSVD) is an intracranial vascular disorder caused by the impairment of cerebral microvessels, with various pathological backgrounds. Currently, a clear and definitive set of diagnostic criteria for CSVD continues to be elusive, and its diagnosis relies primarily on medical history, clinical presentation, brain imaging features, and genetic detection. In clinical practice, no single biomarker was identified. Previous studies have delved into various biomarkers in CSVD and their relationships with cognitive functions, yielding promising candidates. However, drawing a unified and conclusive consensus poses a challenge due to the inherent heterogeneity in research. Therefore, we reviewed the current progression of CSVD biomarkers and their relationship with cognitive functions, mainly including neuroimaging markers and biomarkers related to cerebrospinal fluid (CSF), as well as blood to assist clinicians in diagnosis.

## 1 Introduction

Cerebral small vessel disease (CSVD) is a type of clinical syndrome characterized by varying degrees of damage due to small vessels (including perforating arteries, small veins, arterioles, and capillaries), leading to typical imaging features and pathological alterations (1). As an age-related disease, CSVD affects nearly 100% of individuals aged >90 years, accounting for 45% of dementia cases, 25% of stroke cases, and other central nervous system diseases (2). Lesions induced by CSVD can also result in gait problems, dementia, urinary incontinence, and other related manifestations. Furthermore, CSVD has emerged as the dominant cause of cognitive decline on a global scale, posing a substantial burden to societies (3).

While the significance and concerns surrounding CSVD are well acknowledged, conclusive diagnostic standards remain elusive. The current approach to diagnosis mainly depends on factors such as clinical presentation, laboratory examination, imaging features (4), and, if necessary, genetic detection or pathological biopsy. Evidence has shown that certain biomarkers, such as C-reactive protein (CRP), YKL-40, and homocysteine (HCY), are associated with cognitive outcomes and offer valuable insights into disease activity and progression (5). However, CSVD often remains asymptomatic in many individuals. Brain imaging markers like white matter hyperintensities (WMH) and cerebral microbleeds (CMBs) may be observed in individuals with non-cognitive neurological disorders and in healthy elderly populations, with unknown clinical significance (6). Thus, a deeper understanding of various detectable and favorable biomarkers is crucial to address these diagnostic and treatment challenges and predict prognosis.

## 2 Neuroimaging markers in CSVD

Various features on magnetic resonance imaging (MRI) have been identified as crucial markers of CSVD, encompassing WMH, CMBs, lacunae of presumed vascular origin, perivascular spaces (PVS), recent small subcortical infarcts (RSSI), and cerebral atrophy (7). These neuroimaging markers may manifest independently or concurrently and often exhibit close associations with each other. Researchers also take advantage of advanced imaging techniques to investigate pathological features and predict the progression of diseases. For example, there are existing studies that have used amyloid-positron emission tomography (PET) and tau-PET to detect the pathologies of Alzheimer’s disease (AD) (8). With the assistance of advanced imaging techniques, early identification of pathologic features can be performed, offering valuable guidance to impede disease progression.

### 2.1 White matter hyperintensities (WMH)

WMH is believed to result from chronic brain ischemia, characterized by intensive signals on fluid-attenuated inversion recovery (FLAIR) MRI (9). Several risk factors are well established, especially age and hypertension playing crucial roles in their development. Additionally, cerebral amyloid angiopathy (CAA), atheromatous disease, and certain infections have also been shown to be causes and risk factors for WMH (4). Compared to patients without CAA, those with CAA had a larger WMH burden (8). A wealth of evidence indicates that a higher WMH burden is linked to more severe cognitive impairments in CSVD, particularly affecting processing speed, executive function, and episodic memory (10). Moreover, the unique spatial distribution of WMH can help distinguish between various disease etiologies: juxtacortical WMH are independently associated with CAA, parietal WMH are associated with brain amyloid accumulation, and periventricular WMH are unrelated to any disease phenotype. The spatial patterns of WMH can serve as etiology-specific imaging markers, aiding in the identification of underlying pathological processes and distinguishing between CSVD, CAA, AD, and normal aging (9).

### 2.2 Cerebral microbleeds (CMBs)

CMBs are cardiovascular deposits of blood degradation products contained in macrophages, appearing as tiny, oval, or round lesions in T2-weighted MRI (11). CMBs are not uncommon in all groups of populations and are particularly prevalent in people with CSVD (12). Though often clinically silent, a certain number of CMBs are distributed in specific anatomical regions, resulting in brain tissue damage and functional impairment, leading to cognitive decline, stroke recurrence, and dementia, which means poor outcomes (13).

The specific location of the CMBs has been proven to provide valuable insights into the type of underlying pathology. For instance, hypertensive cardiomyopathy is associated with deep or mixed microbleeds, while the presence of multiple strictly lobar CMBs is one of the imaging markers of CAA (14). In a study, 959 patients were assessed for abnormalities in specific cognitive domains. The findings indicated that cognitive impairments were primarily associated with lobar CMBs, particularly affecting visuospatial deficits and executive functions, whereas similar correlations were not observed in other brain regions (11). Additionally, Li et al. (15) discovered that patients with CMBs exhibited elevated levels of iron deposition in the thalamus, amputate, and hippocampus. To calculate the burden of brain iron deposition specifically for CMBs lesions in each patient, the authors multiplied their mean susceptibility value by the number of voxels involved, obtained from quantitative susceptibility mapping. Notably, their findings suggest that basal ganglia-associated brain iron accumulation may serve as a pivotal factor in driving cognitive deterioration among individuals with CSVD. Furthermore, emphasizing the iron deposition load in the lesions of CMBs may be a quantitative imaging marker of cognitive decline (15). These findings have crucial implications for understanding and diagnosing CSVD and predicting its relationship with cognitive decline.

### 2.3 Perivascular spaces (PVS)

Studies have shown that PVS may indicate a more rapid decline in information processing speed, with no influence on memory or executive function. They also found that higher PVS counts not only increased the potential risk of RSSI and CMBs but also contributed to greater progression of WMH (16). Hilal et al. also pointed out that higher basal ganglia PVS (BG-PVS) counts indicated worse performance on vasomotor speed (17). Furthermore, larger PVS volumes increase the odds of all-cause cardiovascular diseases (CVDs), especially since thalamic PVS is associated with higher odds of transient ischemic attack (TIA). The findings above suggest that, compared to individuals without PVS, those with baseline extensive PVS experience a greater decline in processing speed and show a stronger association with CSVD progression.

### 2.4 Recent small subcortical infarct (RSSI)

RSSI, which is primarily attributed to the occlusion of penetrating arteries, often appears as hyperintensities on T2-weighted MRI and isointense or hypointense on T1-weighted MRI (18). The RSSI is closely linked to WMH (19). Studies have shown that subcortical white matter RSSI more frequently contacts surrounding WMH and occurs in patients with a severe burden of WMH. The observations collectively reflect the cumulative burden of vascular injury and contribute to the overall assessment of disease severity (20). Identifying RSSI on neuroimaging facilitates the timely diagnosis of CSVD. Clinically, patients presenting with RSSI often exhibit a higher risk of recurrent strokes, highlighting its significance as a prognostic marker, making it a critical component in evaluating disease progression and guiding therapeutic strategies.

### 2.5 Other neuroimaging markers

Brain atrophy mostly occurs in people aged >50 years. It involves more than just a reduction in brain volume; it also includes the loss of neurons and their connections, cortical thinning, and subcortical vascular pathology, among others (5). A relatively lower brain volume does not necessarily indicate well-defined macroscopic focal damage, which is typically assumed from a decrease in specific gray or white matter volumes (21). Brain atrophy often coexists with WMH and is linked to cognitive decline. It is suggested that WMH might expedite brain atrophy, positioning it as a risk factor for this degenerative process (21).

Lacunae are considered secondary to fluid-filled glacial cavities, typically measuring 3–15 mm in diameter, and are formed after small cortical infarctions. They exhibit a signal similar to cerebrospinal fluid (CSF) and are predominantly located in the half-oval center and basal node. Reduced local cerebral blood flow resulting from lacunae-induced cerebral perfusion may contribute to cognitive impairments and demonstrative changes. Lacunae have been identified as significant predictors of cognitive decline in patients with CSVD.

**Figure 1:**
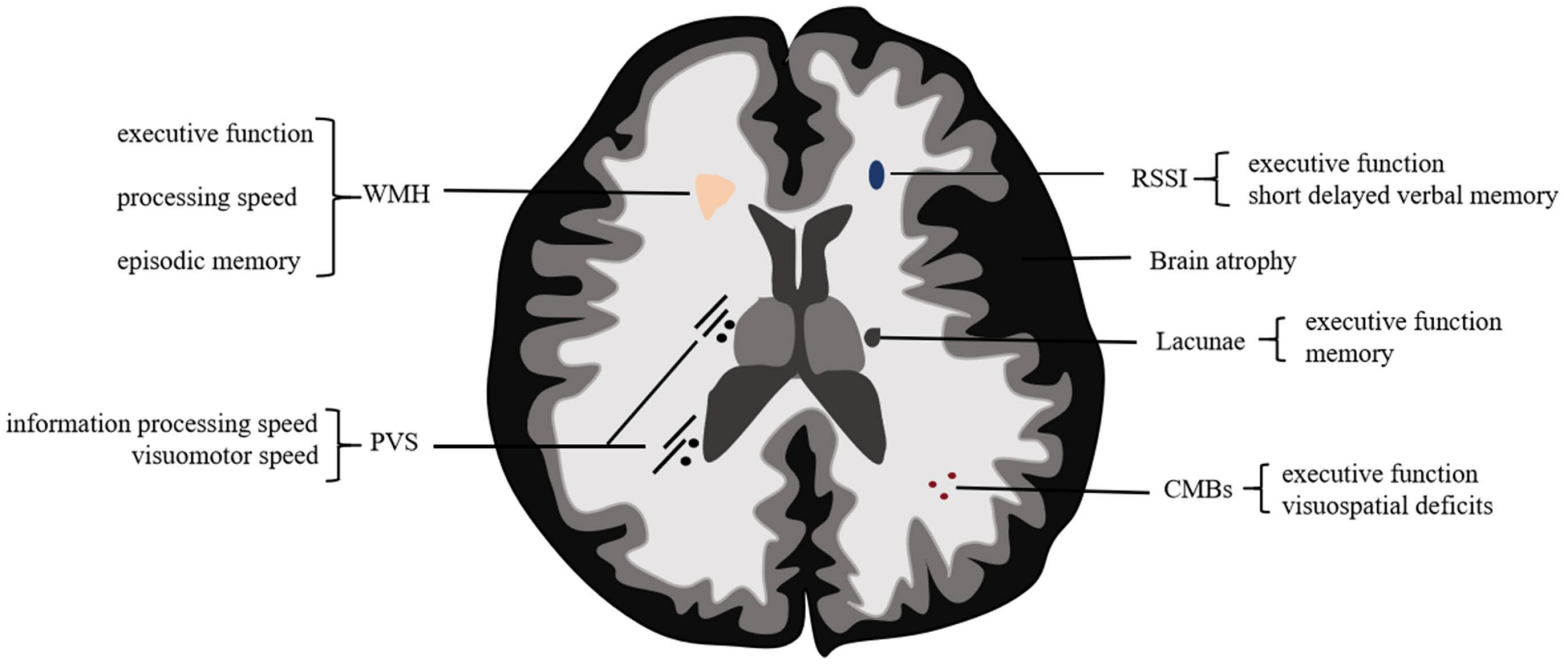
The relationship between neuroimaging markers and cognitive dysfunction in CSVD. Neuroimaging markers of CSVD are closely related to cognitive function, with each marker contributing distinctively to cognitive decline. WMH impairs executive function, processing speed, and episodic memory, while CMBs are associated with declines in visuospatial deficits and executive function. PVS not only affect information processing speed but are also linked to an increased risk of RSSI and CMBs, as well as greater WMH progression. Multiple RSSIs are associated with poststroke cognitive decline, particularly executive function and short delayed verbal memory. Lacunae impairs executive function and memory. Brain atrophy, often coexisting with WMH, leads to global cognitive decline. CSVD, cerebral small vessel disease; WMH, white matter hyperintensities; CMBs, cerebral microbleeds; PVS, perivascular spaces; RSSI, recent small subcortical infarcts.

Overall, the evaluation of neuroimaging markers supports the early detection of CSVD and can serve as a predictor of stroke risk, which is closely related to cognitive decline and stroke recurrence (Figure 1).

## 3 Blood biomarkers in CSVD

Though the use of neuroimaging is an increasingly valuable diagnostic tool, MRI is less frequently used for screening and is usually confined to patients presenting with clinical symptoms or those with established vascular risk factors. Biomarkers are gaining favor among researchers because of their advantages in indicating lesions and predicting outcomes in the very early stages of diseases. Lumbar punctures are relatively invasive procedures that limit the widespread clinical application of CSF biomarkers (22). Therefore, blood biomarkers are increasingly considered reliable indicators of the dynamics of CSVD (Table 1).

**Table 1:** Blood biomarkers of CSVD.

Blood biomarkers	Roles in CSVD	Ref
CRP and hs-CRP	Poor cognitive outcomes; recurrent strokes and other vascular events	(24, 27)
IL-6	Stroke, vascular disease, and cardiovascular death	(24)
YKL-40	White matter damage	(30)
HCY and MTHFR	Cognitive impairment	(33)
Exosomal miRNA-223-3p	Cognitive impairment	(35)
NOS	Brain infarctions and WMH	(38, 39)
TFPI	Progression of WML and LS	(43, 45)
t-PA	Lacunar infarction	(47)
FIB	Radiological progression	(49)
Lp-PLA2 and SOD	Presence of WMH	(50)
Aβ	Cognitive impairment	(63, 65)
BNP and N-T proBNP	Cognitive status	(69, 70)
NOTCH3	Cerebrovascular damage	(72, 76)
Genes related to RAS	Progression of SBI and WMH	(77)
APOE e4	Cerebrovascular damage	(80)

CRP, C-reactive protein; hs-CRP, High-sensitivity C-reactive protein; IL-6, Interleukin-6; HCY, Homocysteine; MTHFR, Methylenetetrahydrofolate reductase; NOS, Nitric oxide syntase; TFPI, Tissue factor pathway inhibitor; t-PA, Tissue plasminogen activator; FIB, Fibrinogen; Lp-PLA2, Lipoprotein-associated phospholipase A2; SOD, Superoxide dismutase; BNP, Brain natriuretic peptide; N-T proBNP, N-terminal pro-brain natriuretic peptide; APOE, Apolipoprotein E.

### 3.1 Inflammatory biomarkers

#### 3.1.1 C-reactive protein (CRP) and high-sensitivity C-reactive protein (hs-CRP)

CRP is an acute-phase protein produced by hepatocytes (23). It has previously been shown to be associated with neurodegenerative diseases and poor cognitive outcomes in normal aging (24). CRP is also considered a marker of inflammation involved in the pathophysiology of CSVD (25), closely related to larger WMH volume, PVS, brain atrophy, and deep CMBs, and is thus significantly associated with greater cognitive impairments in patients with CSVD (24). In the population-based Rotterdam Scan Study of 1,033 individuals of similar age (26), higher plasma CRP levels were significantly associated with the presence and progression of WMH and marginally associated with a greater prevalence of lacunar infarcts. Hs-CRP is more sensitive than CRP. In patients with mild ischemic stroke, the severity of CSVD lesions was positively correlated with serum hs-CRP (27). Hs-CRP levels predict the risk of recurrent strokes and other vascular events and are also relevant to unfavorable outcomes.

#### 3.1.2 Interleukin 6 (IL-6)

Substantial evidence supports the associations between inflammatory biomarkers (IL-6 included) with outcomes such as stroke, vascular disease, and cardiovascular death (28). IL-6 stands out as one of the extensively investigated inflammatory biomarkers concerning the presence and progression of CSVD. In a study involving 1,841 participants conducted by Satizabal et al., the authors observed a cross-sectional association between elevated IL-6 levels and increased total and periventricular WMH volume, with no discernible link between IL-6 levels and deep white matter hyperintensity (DWMH) (24). Therefore, IL-6 plays a crucial role in judging the CSVD severity, predicting prognosis in individuals, and mitigating the risk of CSVD occurrence. It can predict other recurrent vascular events as well.

#### 3.1.3 YKL-40

YKL-40 is a 40 kDa glycoprotein that binds to heparin and chitin. YKL-40 is derived from the single-letter codes of its first three N-terminal amino acids—tyrosine (Y), lysine (K), and leucine (L)—combined with its molecular weight of 40 kDa (29). In the blood, it is considered to be an early biomarker of inflammatory processes (29), and the inflammatory response is one of the pathological mechanisms of CSVD. It is believed to contribute to cognitive decline in CSVD primarily by causing deep white matter damage. This characteristic makes it a potential biomarker for identifying white matter injury. Research has demonstrated that serum YKL-40 levels are higher in patients with CSVD compared to normal cases. In patients with CSVD with cognitive impairments (CSVD-CI), the individual experiences difficulties with mental functions such as memory and thinking, and these impairments are more pronounced in them than in patients with CSVD with no cognitive impairments (30). Thus, serum YKL-40 levels not only offer insights into CSVD-CI but also predict disease progression, making it a promising biomarker for patient prognosis.

### 3.2 Biomarkers of endothelial dysfunction

#### 3.2.1 Homocysteine (HCY) and methylenetetrahydrofolate reductase (MTHFR)

HCY is an amino acid that is produced during the metabolic process of methionine (31). Higher expression of HCY has been extensively studied and linked to an increased risk of cerebrovascular diseases, including CSVD and AD (32). In a prospective cohort study, adult patients with CSVD exhibiting any level of radiological progression, particularly those with lacunae and brain volume loss, displayed elevated levels of HCY (6). Consequently, HCY levels were identified as independent predictors of the progression of lacunar infarcts in patients with CSVD (33). Elevated HCY levels are a significant risk factor for the progression of CSVD and associated cognitive dysfunction (32). Evidence has shown that genetic predisposition, for example, genetic variants in the MTHFR gene, could potentially disrupt normal homocysteine metabolism, leading to an accumulation of homocysteine, which is known to have detrimental effects on the vascular endothelium (2). Additionally, genetic studies have widely acknowledged that the single-nucleotide polymorphism (SNP) C677T elevates serum HCY levels by reducing MTHFR enzyme activity, whereas the impact of A1298C on HCY levels remains inconclusive. A Mendelian randomization study demonstrated that, compared to those without the mutation, individuals with the MTHFR C677T mutation exhibited increased HCY levels and a higher risk of asymptomatic lacunae (34). The observed effects could be attributed to HCY-induced endothelial cell injury and hypoperfusion.

The interplay between HCY, MTHFR polymorphisms, and vascular pathology underscores the need for further research into targeted therapies that address homocysteine metabolism, which could offer significant benefits in the management of CSVD and cognitive impairments. For patients with a genetic background, lowering HCY levels may be crucial in slowing CSVD progression and facilitating the recovery of cognitively impaired patients.

#### 3.2.2 Exosomal miRNA-223-3p

Exosomes are small and uniform membranous vesicles secreted by most cells in the body, carrying various signaling molecules and involved in cell-to-cell communication (35). These vesicles contain various biological molecules, including miRNA, which are crucial for the regulation of numerous physiological processes. One such miRNA, miRNA-223-3p, has been identified as a key regulator of synaptic function and is involved in modulating antigen endocytosis (36). Prior research has highlighted the significance of endothelial-derived exosomes, particularly in relation to cerebral health. These exosomes have been implicated in the damage of cerebral endothelial cells and contribute to the dysfunction of the blood–brain barrier (BBB). Such dysfunction is a notable factor in several neurological conditions (24). Studies have observed that miRNA-223-3p levels are altered in patients with CSVD, particularly those experiencing cognitive impairment. Specifically, elevated levels of miRNA-223-3p are associated with increased levels of HCY and CRP, two biomarkers commonly associated with inflammation and vascular damage (35). In the context of CSVD, it was found that the levels of miRNA-223-3p were significantly higher in patients with cognitive impairment compared to those with CSVD alone. This finding indicates a positive correlation between miRNA-223-3p levels and both HCY and CRP levels, indicating that, as cognitive impairment in CSVD progresses, the expression of miRNA-223-3p increases.

Given these findings, miRNA-223-3p exhibits promise as a biomarker for tracking cognitive decline in individuals with CSVD. The association of CSVD with both inflammatory markers and cognitive impairment highlights its possible role in the pathophysiology and offers a promising avenue for further research into diagnostic and therapeutic strategies for this condition.

#### 3.2.3 Nitric oxide synthase (NOS)

NOS is responsible for the production of nitric oxide (NO), a molecule with important vasodilatory and anti-inflammatory properties. Endothelium-derived NO signaling plays a role in vasculogenesis during the early developmental stage (37). Genetic polymorphisms in the NOS gene could affect the production and function of nitric oxide, leading to alterations in vascular tone and increased susceptibility to oxidative stress, both of which have been implicated in the development of brain infarctions and WMH (38, 39). A study showed that endothelial NOS-deficient mice spontaneously develop multiple hypoperfused or occluded areas. Moreover, these mice also display microinfarctions, microbleeds, and cerebral amyloid angiography (37). In addition, several other endothelial biomarkers were also studied. Von Willebrand factor (vWF) is synthesized in endothelial cells and megakaryocytes. Higher levels of vWF reflect activation of the endothelial system during a stroke, while a lack of vWF expression indicates impaired cerebral endothelial function (23). Asymmetric dimethylarginine (ADMA) is an endogenous NOS inhibitor. Elevation of ADMA was associated with MRI findings of small vessel disease, especially with leukoaraiosis and serious bacterial infection (SBI) (40, 41). Elevated intracellular adhesion molecule 1 was associated with WMH progression (42).

### 3.3 Coagulation and fibrinolysis biomarkers

#### 3.3.1 Tissue factor pathway inhibitor (TFPI)

TFPI serves as the primary physiological inhibitor of tissue factor (TF)-induced coagulation, playing a pivotal role in maintaining hemostatic balance. Its anticoagulant effect is mediated by binding to factor Xa within the TF-VIIa/Xa complex, thereby inhibiting thrombin generation. Beyond its canonical role in coagulation, evidence underscores the significance of TFPIs in the pathogenesis of CSVD, particularly in white matter lesion (WML) and lacunar stroke (LS).

Studies have demonstrated that an imbalance in the TF-TFPI axis may contribute to microvascular pathology in CSVD. Specifically, the severity of WML has been shown to be positively correlated with the ratio of TF to TFPI, suggesting that dysregulation of coagulation pathways exacerbates WML progression (43). These findings indicate that reduced TFPI may exacerbate the prothrombotic environment and endothelial dysfunction, promoting microvascular damage in WML.

LS is primarily caused by increased BBB permeability resulting from endothelial cell activation (44). In a study comparing 149 patients with LS with 42 controls, patients with LS showed elevated levels of full-length (FL) TFPI (the mature form of TFPI), especially those classified as having isolated lacunar infarctions (ILA) based on MRI findings. After adjusting for confounding factors like age and LDL cholesterol, higher FL TFPI levels were observed in ILA patients compared to controls (45). The increased FL TFPI in LS patients suggests that endothelial dysfunction plays a critical role in its pathogenesis.

#### 3.3.2 Tissue plasminogen activator (t-PA)

t-PA is a member of the serine protease family. Its primary physiological role lies in the breakdown of fibrin deposits, thereby facilitating the removal of intravascular fibrin buildup and maintaining vascular patency. However, t-PA also has notable implications for BBB dynamics, which is a critical factor in the pathogenesis of CSVD (46). Experimental studies using animal models have highlighted the dual role of t-PA in neuroprotection. While its expression diminishes with aging, particularly in white matter regions, its presence is associated with reduced white matter ischemic damage. This finding is especially pertinent to CSVD, where white matter lesions are a key clinical feature (47). Notably, decreased levels of t-PA have been linked to lacunar infarctions, a subtype of ischemic stroke frequently observed in patients with CSVD.

Collectively, t-PA plays a multifaceted role in cerebrovascular health, influencing both the vascular and parenchymal components of the brain.

#### 3.3.3 Fibrinogen (FIB)

FIB is a large plasma glycoprotein synthesized in the liver and plays a central role in coagulation and hemostasis (48). Within the brain, FIB and its breakdown products are removed through the local t-PA/plasminogen system, emphasizing its integration into neurovascular processes. Elevated levels of FIB or its incomplete clearance may contribute to vascular and parenchymal damage (40). It has been reported that FIB was independently associated with an increased risk of radiological progression (new lacunae or WMH progression), regardless of the clinical CSVD manifestation (49).

### 3.4 Lipoprotein-associated phospholipase A2 (Lp-PLA2) and superoxide dismutase (SOD)

In a retrospective study involving 87 patients with CSVD, researchers found an association between the inflammatory marker Lp-PLA2 and the anti-inflammatory enzyme SOD with WMH burden (50). Specifically, Lp-PLA2 is a pro-inflammatory molecule closely linked to cardiovascular and cerebrovascular risks (51), whereas SOD plays a role in mitigating oxidative stress by catalyzing the conversion of superoxide radicals into hydrogen peroxide and oxygen, thus exerting anti-inflammatory effects (52).

The study analyzed enzyme levels in patients with varying degrees of WMH, revealing that patients with severe WMH had lower levels of Lp-PLA2 and SOD compared to those with mild WMH. This finding suggests that low levels of Lp-PLA2 and SOD may be associated with the severity of white matter lesions and may also be associated with cognitive impairment in CSVD (50). Previous research has explored the crucial relationship between cognitive dysfunction and white matter lesions (10), and this study further indicated that this relationship may be mediated by the interplay between inflammation and oxidative stress. Moreover, SOD levels were positively correlated with the neuroinflammatory marker YKL-40, indicating a potential interaction between neuroinflammation and oxidative stress (29). The correlation between SOD and YKL-40 implies that inflammation and oxidative stress are two interrelated mechanisms in the pathogenesis of CSVD.

By simultaneously monitoring these two enzymes, a better understanding of pathological processes in patients can be achieved. This joint analysis approach may help to more accurately assess the severity of CSVD and the risk of cognitive dysfunction, thereby providing stronger support for personalized therapeutic interventions.

### 3.5 Neurofilament light chain (NfL)

NfL is a structural protein abundant in neuronal axons that is released into bodily fluids upon axonal damage (53). Serum neurofilament light chain (sNfL) is a biomarker of neuronal injury, including damage associated with CSVD. Studies have shown that cerebrovascular pathology affects sNfL. Elevated serum levels were observed in patients with an RSSI and in patients with stroke (54, 55). NfL levels demonstrated a strong association with impairments in processing speed performance in patients with CSVD (56). Moreover, high sNfL concentrations are linked to worse cognitive performance and increased risk of functional decline, making them a valuable indicator of disease progression. Furthermore, lacunae or brain volume showed a weaker association with processing speed (56), suggesting that serum NfL might have equal or even greater utility than these conventional MRI markers. In fact, beyond its implications in CSVD, investigations have unveiled heightened sNfL levels in trauma, AD, multiple sclerosis (57), and cerebrovascular disease (58). Therefore, sNfL is not specific for a particular pathology.

Although CSF NfL has been widely used as a biomarker of neuronal damage, its collection via lumbar puncture is invasive and less feasible for routine monitoring. Hence, sNfL is a minimally invasive alternative to blood sampling. Importantly, advancements in ultrasensitive detection techniques, such as the single molecule array (Simoa) assay, have enabled the reliable measurement of sNfL in serum at concentrations previously undetectable (59). This breakthrough in Simoa assays not only mitigates the invasiveness associated with lumbar punctures but also broadens the scope of clinical applications, offering valuable insights into various neurological conditions.

### 3.6 Amyloid-*β* (Aβ)

Aβ with their varying forms playing distinct roles in the pathogenesis of vascular conditions (60). Aβ42 and Aβ40 are the most widely studied. Aβ42 is known to aggregate into senile plaques, a hallmark of the disease, whereas Aβ40 has been implicated in cerebrovascular amyloid deposition, notably in conditions like CAA (61, 62). The link between AD and vascular pathology has become a critical focus in neurology, as increasing evidence suggests that cerebrovascular dysfunction may contribute to cognitive decline and neurodegeneration. A key study that supports this connection is the Rotterdam study, which pointed out that elevated levels of Aβ40, along with a higher Aβ40/Aβ42 ratio, were associated with cognitive impairment in CSVD (63). Their findings underscore the importance of Aβ40 in vascular-related cognitive decline, suggesting that amyloid pathology extends beyond the traditional boundaries of AD.

Further supporting the role of Aβ biomarkers in neurodegeneration, Udeh-Momoh et al. conducted a study involving 115 AD patients (64). Their research demonstrated that the Aβ42/Aβ40 ratio was a strong predictor of amyloid positivity, as determined by PET scans. This ratio has the potential to serve as a blood-based biomarker for early AD detection (64), which indicates that the balance between different Aβ species may be crucial for identifying individuals at risk of amyloid-related neurodegeneration. Additionally, a study by Qu et al. introduced a novel sandwich ELISA (enzyme-linked immunosorbent assay) designed to detect various soluble Aβ42 species, including monomers, oligomers, and total Aβ42 (65). Their results showed that oligomeric Aβ42 (oAβ42) and total Aβ42 (tAβ42) levels were promising biomarkers for CSVD and its associated cognitive impairment. This assay adds a new dimension to the potential diagnostic tools available for detecting amyloid pathology in vascular-related cognitive decline.

Overall, these findings suggest that blood levels of Aβ, particularly the Aβ42/Aβ40 ratio and specific soluble Aβ species, are strongly correlated with the severity and progression of CSVD. As research advances, these biomarkers may prove valuable not only for tracking central nervous system damage in patients with CSVD but also for predicting cognitive decline and aiding in the early detection of AD. These insights reflect the growing recognition of the interplay between amyloid pathology, vascular health, and cognitive function.

### 3.7 Brain natriuretic peptide (BNP) and its precursor fragment N-terminal pro-brain natriuretic peptide (N-T proBNP)

Recent studies have increasingly highlighted the association between cardiac microvascular dysfunction and CSVD because cardiovascular diseases confer an increased risk of CSVD and its sequelae, such as cognitive impairment (66). BNP and its precursor fragment N-T proBNP are regarded as sensitive indicators of cardiac dysfunction and heart failure. However, with the deepening of research, it has been discovered that the elevation of their levels is also significantly associated with the occurrence and development of cerebrovascular diseases (67). The potential indirect mechanisms are rather complex, mainly involving cerebral hypoperfusion, cardioembolic stroke, and ischemic conditions caused by atherosclerosis, among other aspects (68). Specifically, an increase in the level of N-T proBNP is closely related to an elevated risk of stroke and subclinical cerebrovascular lesions, such as silent cerebral infarction. In a further study, higher baseline plasma levels of NT-proBNP and hs-cTnT were associated with an increased risk of cognitive decline and showed distinct associations with CSVD progression (69). A population-based study indicated a significant correlation between the elevation of plasma BNP levels and lacunar infarction in patients with ischemic CSVD (70). In addition, Vilar-Bergua et al. also found that a higher level of NT-proBNP was independently associated with CMBs, PVS, and WMH volumes (71). The possible mechanism is that NT-BNP reduces local blood flow and blood pressure, thereby diminishing cerebral blood flow and causing ischemic injury.

### 3.8 Gene markers

#### 3.8.1 NOTCH3

Cerebral Autosomal Dominant Arteriopathy with Subcortical Infarcts and Leukoencephalopathy (CADASIL) is a monogenic form of CSVD caused by mutations in the NOTCH3 gene. It is the most common familial CSVD (72). Pathological changes in the pial and small perforating cerebral arteries are an important feature. Typical microvascular changes occur throughout the arterial tree. After the gene mutation, the NOTCH3 protein becomes fragmented, and its end products accumulate at the cell membrane of vascular smooth muscle cells, eventually leading to the destruction of the vascular smooth muscle cells (73). In addition to affecting the cerebral vessels, genetic mutations also induce pathological changes in the cardiovascular system. It has also been observed in experimental animal models (74) and in humans (75). Genetic research has further confirmed the inherent connection between cerebral and cardiac small vessel diseases. The vascular smooth muscle cells, which control coronary vascular resistance and adjust coronary perfusion, that degenerate in CADASIL. As a result, the vascular tone and autoregulatory response of microvessels may be severely compromised, which could lead to a diminished vasodilator reserve and, hence, to increased susceptibility to ischemia and myocardial infarction (76). Therefore, NOTCH3 mutation carriers may be more vulnerable to the deleterious effects of classical cardiovascular disease risk factors.

#### 3.8.2 Renin-angiotensin system (RAS)

Polymorphisms in key genes related to RAS, such as the angiotensin-converting enzyme gene (ACE), the angiotensinogen gene (AGT), and the angiotensin II type 1 receptor gene (AGTR1), have been implicated in various cardiovascular and cerebrovascular processes, and their genetic polymorphisms might play a crucial role in the progression of SBI and WMH (77).

#### 3.8.3 Apolipoprotein E (APOE)

APOE is a critical genetic factor influencing lipid metabolism and cerebrovascular health. Certain APOE alleles may promote lipid accumulation in the brain, thereby exacerbating cerebrovascular damage. APOE e4 carriers exhibit increased amyloid deposition, which impairs clearance mechanisms reliant on perivascular arteriolar pulsations. Variations in APOE alleles, particularly APOE e4, have been implicated in the pathogenesis of cerebrovascular lesions. The APOE is closely associated with AD. The APOE e4 allele is the strongest genetic risk factor for sporadic AD (78) and an independent predictor of AD diagnosis (79).

A population-based study provided evidence supporting a correlation between PVS and traditional vascular risk factors in older adults (80). Orthostatic hypotension reduces the flow velocity in the middle cerebral artery, leading to brain hypoperfusion. This hypoperfusion impairs the clearance of amyloid through the PVS, which relies on arteriolar pulsations for efficient metabolite removal (78). Subsequently, amyloid deposition increased. Such impaired clearance mechanisms may explain the higher PVS burden observed in individuals with orthostatic hypotension. Notably, the association between orthostatic hypotension and lobar PVS is particularly pronounced among APOE e4 carriers. This association indicates that lobar PVS could serve as a marker for amyloid-associated small vessel disease, thereby linking vascular dysfunction and amyloid pathology in this population.

A pooled analysis of multiple cohort studies by Tavia et al. explored the determinants of PVS in various brain regions, including the mesencephalon, hippocampus, basal ganglia, and centrum semiovale (81). The study found a significant association between APOE genotypes and PVS. APOE e4 carriers demonstrated the strongest correlation with increased PVS counts in the hippocampus (odds ratio: 1.03 [1.01–1.05]) (81). The analysis highlights the region-specific effects of APOE e4 on PVS. APOE e4 plays a dual role in promoting both amyloid pathology and cerebrovascular dysfunction, with significant implications for CSVD. The allele’s association with PVS, particularly in the hippocampus, underscores its potential as a marker of CSVD.

## 4 Cerebrospinal fluid biomarkers in CSVD

In addition to neuroimaging markers and blood biomarkers, CSF biomarkers play a critical role in understanding CSVD. CSF can directly contact the brain and the spinal cord and is the main carrier of metabolites and pathological changes in the central nervous system (CNS). Therefore, CSF biomarkers can more accurately reflect pathological changes in the nervous system, such as neuronal damage, inflammation, and abnormal protein aggregation. Despite the relatively invasive nature of collecting CSF, its advantages in early diagnosis of disease, pathological monitoring, and treatment assessment are irreplaceable.

### 4.1 NfL

NfL is primarily expressed in large-diameter nerve axons. It is first released into the extracellular space and subsequently enters the CSF and blood when the axons undergo damage (58). Thus, the levels of NfL in body fluids can serve as indicators of pathological changes related to brain damage, brain atrophy, and a wide spectrum of neurological diseases (82). It has been observed that CSF NfL levels may be linked to certain imaging features of CSVD, particularly with WMH and RSSI (53). Huss et al. revealed that, compared to patients with mild or moderate WMH, those with severe WMH exhibited higher levels of NfL (22). The findings are consistent with previous studies (56). In summary, in the context of elderly individuals affected by the development of CSVD, elevated CSF NfL levels can assist in identifying the severity of CSVD.

### 4.2 Glial fibrillary acidic protein (GFAP)

GFAP is an intermediate filament protein that has been identified as a highly promising body fluid marker for CSVD (83). In a prospective study, patients with CSVD with elevated serum levels of GFAP (sGFAP) and NfL (sNfL) were found to exhibit higher modified Rankin Scale (mRS) scores and National Institutes of Health Stroke Scale (NIHSS) scores, indicating a significant correlation between serum biomarker levels and cognitive function (22). Furthermore, the detection values of GFAP and NfL in cerebrospinal fluid samples showed analogous results compared with serum samples. It is reasonable to believe that elevated levels of CSF GFAP also significantly correlate with clinical severity and cognitive dysfunction, suggesting a poor prognosis for individuals with CSVD.

Both the CSF and serum levels of GFAP have been studied as potential biomarkers for CSVD and are linked to disease severity and progression in CSVD (84). However, there are important differences between them. CSF GFAP is believed to more directly reflect CNS injury, as it crosses the BBB during pathological events. In contrast, serum GFAP levels may be influenced by peripheral sources, making them less specific to CNS pathology.

Elevated CSF GFAP levels are associated with more severe forms of CSVD and cognitive decline, whereas sGFAP, which shows promise as a less invasive alternative, may not be as precise in capturing CNS-specific changes. Thus, CSF GFAP may hold greater prognostic value for long-term outcomes in CSVD, whereas sGFAP could be useful for monitoring disease progression in a more accessible manner. Future research should aim to further delineate the relative utility of these biomarkers and explore their combined use for improved diagnostic and prognostic assessment of CSVD.

### 4.3 Amyloid-*β* (Aβ)

Aβ peptides are key byproducts of the processing of amyloid precursor proteins, and they contribute to generalization upon reaching a certain threshold (60). In conditions like AD and CAA, elevated plasma Aβ levels are commonly observed due to increased Aβ production and impaired Aβ clearance. Interestingly, a majority of patients with CSVD display normal levels of Aβ40 and Aβ42 (85). In a study comprising 88 patients with probable AD, the presence of CAA-related cortical microbleeds was found to be associated with CSF levels of Aβ, primarily comprising Aβ40 and Aβ42. The observed decline in CSF Aβ40 and Aβ42 levels reflects the deposition of these amyloid proteins in the cerebrovascular system (86). The findings highlight the significance of CAA-related pathology in influencing the dynamics of Aβ deposition and further deepen our understanding of the complex interaction between amyloid pathology and cerebrovascular manifestations in AD.

In addition, a separate study demonstrated a correlation between cortical microbleeds and a low ratio of CSF Aβ42/Aβ40. CSF levels of Aβ40 were found to be strongly correlated with CSVD with diffuse cardiomyopathy who had experienced acute stroke (87). Another investigation highlighted the association between Aβ40 and both WMHs and lacunar infarcts in individuals with AD and those with CAA. The combined evaluation of imaging markers, CSF Aβ levels, and Aβ42/Aβ40 ratio offers a novel avenue for the diagnosis and differential diagnosis of CSVD. This integrated approach holds promise in providing a more comprehensive understanding of the complex relationships between imaging and biochemical markers in the context of CSVD diagnosis.

### 4.4 Tau

Neurofibrillary tangles (NFT) composed of hyperphosphorylated tau and senile plaques formed by Aβ are hallmark pathological features of AD (88). Tau, Aβ, and CSVD occasionally coexist. Emerging evidence suggests a potential link between tau pathology and CSVD. Kim et al. used 18F-AV1451 PET imaging to assess the role of tau in a cohort of patients with superior vena cava isolation (SVCI). AV1451 is a ligand for paired helical filament tau and is able to detect NFT *in vivo*. Advanced neuroimaging techniques revealed that a higher burden of CSVD is correlated with increased tau deposition, particularly in the inferior temporal regions of the brain. Notably, this relationship appears to persist regardless of Aβ positivity (89). It is suggested that tau accumulation in the context of CSVD may occur independently of classic amyloid pathology. Additionally, ischemic events may exacerbate tau pathology by increasing tau accumulation and promoting hyperphosphorylation. Animal studies have demonstrated that chronic cerebral hypoperfusion enhances tau hyperphosphorylation (90), and impaired blood flow or microvascular damage is associated with increased tau aggregation (91). This finding highlights a possible mechanism through which CSVD, via ischemia, contributes to the neurodegenerative processes (92). These findings provide a mechanistic basis for the interplay between CSVD and tau pathology, suggesting that cerebrovascular dysfunction may play a pivotal role in driving tau-related neurodegeneration.

Tau may serve as a promising biomarker for CSVD. Further research is warranted to clarify these connections and explore their implications for the prevention and treatment of cognitive decline in patients with CSVD.

### 4.5 Matrix metalloproteinases (MMPs)

MMPs belong to a large family of structurally related zinc-dependent electrolytic enzymes involved in the process of digesting components of extracellular matrix components, remodeling neurons, and sustaining BBB integrity (23).

Among these, MMP-9 is implicated in several disease processes, including cancer metastasis and psychiatric disorders. In the context of ischemic and hemorrhagic stroke, MMP-9 has been consistently observed to be elevated within 2 h of onset in both the infarct region and penumbra (93). Compared to those without remarkable abnormalities in brain MRI, those with higher levels of CSF MMP-9 tend to experience a more severe executive capacity in ischemic patients with CSVD. There is an association between detectable MMP-9 and WMH in community-based samples (94). It has been found that MMPs are related to the progression of imaging markers, such as WMH and lacunar infarction, suggesting that MMPs may show some promise in the future (Table 2).

**Table 2:** CSF biomarkers in CSVD.

CSF biomarkers	Roles in CSVD	Ref
NfL	Impairments in processing speed and performance	(56, 82)
GFAP	Cognitive dysfunction	(22)
Aβ	Diagnosis and differential diagnosis	(87)
Tau	Cognitive status	(95)
MMPs	Executive capacity	(96)

NfL, Neurofilament light chain; GFAP, Glial Fibrillary Acidic Protein; Aβ, Amyloid-β; MMPs, Matrix metalloproteinases.

## 5 Conclusion

CSVD has emerged as a hot topic recently because of its insidious onset and high incidence rate. It not only serves as a prevalent risk factor for stroke recurrence but is also intricately linked to cognitive impairment. This comprehensive review aims to shed light on the advancements in understanding biomarkers associated with CSVD and their relationship to cognitive dysfunction. We have highlighted several key biomarkers, including neuroimaging markers, cerebrospinal fluid biomarkers, and blood-based markers, each contributing valuable insights into CSVD pathophysiology. Notable imaging biomarkers, including WMH and CMBs, provide critical information on the disease burden and its correlation with cognitive decline. Moreover, NfL, MMPs, and GFAP offer insights into the structural alterations underlying CSVD. However, given the complexity of the pathological mechanisms involved, numerous markers warrant further exploration. Future research should focus on the predictive value of these biomarkers in CSVD and their potential application in improving early diagnostic and therapeutic strategies.
